# Women who live in mining on the French-Brazilian border: daily challenges

**DOI:** 10.1590/0034-7167-2021-0688

**Published:** 2022-08-22

**Authors:** Lise Maria Carvalho Mendes, Flávia Gomes-Sponholz, Juliana Cristina dos Santos Monteiro, Ana Karina Bezerra Pinheiro, Nayara Gonçalves Barbosa

**Affiliations:** I Universidade Federal do Amapá. Macapá, Amapá, Brazil; II Universidade de São Paulo. Ribeirão Preto, São Paulo, Brazil; III Universidade Federal do Ceará. Fortaleza, Ceará, Brazil

**Keywords:** Mining, Border Health, Woman’s Health, Delivery of Health Care, Comprehensive Health Care., Minería, Salud Fronteriza, Salud de la Mujer, Atención a la Salud, Atención Integral de Salud., Mineração, Saúde na Fronteira, Saúde da Mulher, Atenção à, Saúde, Assistência Integral à, Saúde.

## Abstract

**Objectives:**

to describe the daily life of Brazilian women who work in mining.

**Methods:**

a descriptive, qualitative study, with analysis based on the theory of Symbolic Interactionism. Non-participant observations, field diary writing, sociodemographic form, non-directive interviews, recorded and transcribed in full, were carried out with 19 women who work in mining areas on the French-Brazilian Amazon border.

**Results:**

two categories emerged: Life trajectories: women’s work in mining; Woman, mother and prospector: the multiple facets of gender inequality on the Amazon border.

**Final Considerations:**

assessing the daily experiences of women in the Amazonian mines allowed identifying their health needs, evidencing the need to direct and implement public and social policies and health practices for comprehensive care of these women’s health.

## RESUMO

**Objetivos:**

descrever o cotidiano de mulheres brasileiras que exercem atividades laborais em garimpos.

**Métodos:**

estudo descritivo, qualitativo, com análise baseada na teoria do Interacionismo Simbólico. Foram realizadas observações não participantes, tessitura de diário de campo, formulário sociodemográfico, entrevistas não diretivas, gravadas e transcritas na íntegra com 19 mulheres que trabalham em áreas de garimpos na fronteira amazônica franco-brasileira.

**Resultados:**

duas categorias emergiram: *Trajetórias de vidas: a lida das mulheres no garimpo; Mulher, mãe e garimpeira: as múltiplas facetas da desigualdade de gênero na fronteira amazônica*.

**Considerações Finais:**

a avaliação das vivências cotidianas das mulheres no garimpo amazônico permitiu a detecção de suas necessidades de saúde, evidenciando a necessidade de direcionamento e efetivação de políticas públicas e sociais e práticas de saúde para a atenção integral à saúde dessas mulheres.

## RESUMEN

**Objetivos:**

describir el cotidiano de las mujeres brasileñas que trabajan en la minería.

**Métodos:**

estudio descriptivo, cualitativo, con análisis basado en la teoría del Interaccionismo Simbólico. Se realizaron observaciones no participantes, redacción de diario de campo, formulario sociodemográfico, entrevistas no directivas, grabadas y transcritas en su totalidad, con 19 mujeres que trabajan en áreas mineras en la frontera franco-brasileña amazónica.

**Resultados:**

surgieron dos categorías: Trayectorias de vida: el trabajo de las mujeres en la minería; Mujer, madre y prospectora: las múltiples facetas de la desigualdad de género en la frontera amazónica.

**Consideraciones Finales:**

la evaluación de las experiencias cotidianas de las mujeres en las minas amazónicas permitió detectar sus necesidades de salud, evidenciando la necesidad de orientar e implementar políticas públicas y sociales y prácticas de salud para el cuidado integral de la salud de estas mujeres.

## INTRODUCTION

The border between Brazil and French Guiana, an overseas territory of France, is one of the most difficult Brazilian regions to access, in which women’s health conditions are almost completely unknown^(1)^. The discovery of gold in this area, in 1855, radically changed the population arrangement of the surroundings and the activities that sustain the region^(1)^. Even today, this border has a high concentration of gold ore, a specific condition for attracting migrants and that drives the formation of mining in the locality^(2-4)^. It is estimated that there are around 8,000 illegal miners and miners working at approximately 600 gold mining sites in the French Amazon rainforest^(5)^. The location allows the continuity of the gold rush that gives rise to clandestine mining, places where deviations are punished with violence and where girls and women are the most vulnerable^(6-7)^.

In this context, the community of Ilha Bela stands out, an entrepot district between Oiapoque and the Sikini mining site, in French territory, which currently serves as the main logistical support point for mining. It is the place where Brazilians dock to wait for the opportune moment to enter the clandestine mining of the French Amazon^(3)^. Ilha Bela is a riverside community, surrounded by the waters of the middle Oiapoque river, about eight hours by *catraia* (motorized boat) from the city of Oiapoque and which, through operation, driven by mining activities, has a floating population^(3)^. The region is also characterized by high population mobility, despite difficult access due to logistical, administrative and security issues^(2-4)^.

Women are also included among the workers who migrate to mining areas, and, for a long time, the issues inherent to women in mining have been silenced and portrayed in an appendix to gold mining activities^(7)^. In addition to being a woman in a mining environment, gender inequalities, stigma and clandestinity are exacerbated, associated with the geographic isolation of the region in question, which increases the vulnerability of women who carry out any type of activity in these mining activities.

Evidence points to the mining region as one of the most dangerous for men, women and the community, due to a range of chemical, physical, biological, biomechanical and psychosocial hazards, often resulting in rapid development of disease and premature death^(8-9)^. The intense and disordered cross-border population mobility, associated with the difficulty of access to the region by health teams and the persistent incursion of prospectors and prospectors into the forest, favors the rapid spread of diseases^(1-2,5-7)^. Potential health problems include influenza A^(2,10)^, malaria^(2,4,10)^, beri beri^(2,10-11)^, leishmaniasis^(2)^, syphilis^(2,10)^ and other infections. Furthermore, problems can be magnified by the combination of remote locations and lack of health services, which often prevent timely treatments^(12)^. When entering women’s health, current knowledge is limited^(13)^. Women in the mining area constitute a vulnerable group, considering the intersection between gender inequalities, stigma, clandestinity, associated with the geographic isolation of the region in question. These factors increase women’s risk of health problems, such as sexually transmitted infections (STIs), unwanted pregnancies, among others, which expose weaknesses related to gender and sexual and reproductive health^(5,8-15)^.

Most studies address Acquired Immunodeficiency Virus (AIDS) infection, mainly in Africa, and discuss epidemiological aspects, such as marital status, alcohol and drug use, history of previous STIs, partnership or sex between prospectors, multiple sexual partners, pay-for-sex, transactional sex, and non-consistent condom use^(9,14-15)^. Only a few health studies have specifically investigated vulnerable population groups such as women of childbearing age and children^(16-17)^. As can be seen, there are still many gaps regarding the problems that affect Brazilian women in the mining area, specifically in the border in question^(2,18-20)^.

In this way, considering the commitments of the 2030 Agenda and the Sustainable Development Goals, which have, among the priority goals and established policies, the promotion of women’s health and gender equality^(16)^, and understanding that social actors judge aspects of the moment of social interaction of the groups in which they are allocated, such as the mining environment, the interest in carrying out this study arose.

## OBJECTIVES

To describe the daily life of Brazilian women who work in mining.

## METHODS

### Ethical aspects

The research was approved by the Research Ethics Committee. Participants signed the Informed Consent Form (ICF) prior to the beginning of data collection. Statements were identified through the letter I, in numerical order of interviews, to protect anonymity.

### Study design and theoretical-methodological framework

This is a descriptive study, with a qualitative approach, whose analysis process was based on the Symbolic Interactionism (SI) theoretical framework^(17)^. The theoretical perspective of SI thinks the social relationship from the moment of interaction, i.e., the social rules of interaction are arranged at the moment it takes place and interfere with individual behavior, influencing compliance with health care practices^(21)^.

### Methodological procedures

The Consolidated Qualitative Research Reporting Criteria (COREQ) guidelines were used. Participants were selected using the snowball technique, by exponential sampling^(22)^, used to reach groups of difficult access. The inclusion criteria were to be Brazilian, female and to experience the work routine in clandestine mining in the referred border. All women invited agreed to participate in the research^(23)^. The immersion in the field lasted 15 days, and the collection ended when the data began to repeat itself, showing saturation, and new meanings were no longer added. In total, 20 women were included and interviewed. One participant was excluded due to the noise of *catraias* in the audio recording, which made transcription impossible, since, on the day after transcription, the participant had left for mining.

Prior to data collection, a pilot study was carried out to verify the clarity, understanding and ordering of the guiding questions, as well as the best time to approach. There was no need for adjustments. Data collected in the pilot study were excluded from the survey. Pilot study participants also signed the ICF.

### Study setting

Recruitment took place in Ilha Bela, a resting place for prospectors in the region^(3)^. Recruitment in this area is justified, because the mining areas are accessible only through flights allowed by the French authorities or illegally, through long journeys on foot and/or canoe. In view of these logistical and legal limitations and the researcher’s safety, it was decided to recruit in that location. Collection took place in April 2018.

### Data collection, organization and analysis

The process for qualitative data collection, organization and analysis was carried out in phases, being an observation phase, an interview phase and four phases of data analysis. Four enablers were used to obtain data: a) application of a sociodemographic form, prepared by the authors; b) non-participant observation, which focuses on the symbolic communication of participants in their social interaction; c) registration in a field diary; d) conducting a non-directive interview, in a reserved environment, which took place in the ravines - how women refer to their residences - made of wood and asbestos roof, with a single bathroom arranged for all passers-by on the island. These collection techniques were not fixed and were interspersed with each other, occurring concomitantly.

In the first phase of observation, one of the researchers contacted the community leader of Ilha Bela, in order to facilitate the insertion with the community. Data were written daily in a field diary, simultaneously to the first phase of analysis of what was said and observed, which involves postures, gestures, silences, laughter, crying, political, moral and religious values. Writing in the field diary and reflecting on the experience also occurred concomitantly with the transcription of the interviews in full, which made it possible for the raw material to be reviewed and it was possible to resolve doubts that arose during the transcriptions.

The interviews were conducted by the main author of this article, a nurse and professor at a Brazilian higher education institution, with experience in conducting and analyzing qualitative research. The same were recorded in the form of audio, with an average duration of 45 minutes, from the following triggering questions: tell me a little about how you got to work in mining and what it is like to live in mining, particularly the parts that refer to being a woman in this place and which places you go when you need health care. Sociodemographic and sexual and reproductive history data were collected using a structured form.

With the transcribed material, reflective reading began, which enabled the synthesis of data, according to the identification of common or related elements around a concept capable of representing them. Categorizations were performed manually by two researchers. A third researcher was consulted when differences were found in the identification of the most prominent themes. This phase required reflection, critical and creative data analysis. The theme was reflected with themes arranged in the literature, without the intention of exhausting the theme.

## RESULTS

Among the participants, the age group between 30 and 39 years old predominated (7/36.84%). Regarding origin, it was observed that most women came from the North (11/57.89%) and Northeast (6/31.57%) regions. Women who declared themselves to be black and brown (13/68.42%) formed the predominant group, juxtaposed to a profile of low education, in which women who were never literate (6/31.57%) or had not completed elementary school (6/31.57%) formed the majority group. However, one woman was found who had completed higher education (1/5.26%). Some participants reported being bilingual and claimed to understand French, Creole spoken in some places in French Guiana, in addition to Portuguese (4/21.05%). All women reported being cisgender, predominantly heterosexual women (18/94.73%), with a fixed partnership in the last three months (12/63.15%), especially with workers who worked in mining in the region (14/73.68%). The predominant marital status was that of a stable union, in which women reported living together (14/73.68%). The establishment of intimate partnerships with foreigners was considered by them as a mechanism for social ascension in the mining community.

They were younger than 15 years old when they lost their virginity (19/100%). One of them reported that her first sexual relationship was a rape perpetrated by her boss, while she was working as a domestic and nanny before migrating to work in mining. As for parity, women with children predominated (17/89.47%). Of these, half reported having experienced at least one spontaneous and/or induced abortion (10/52.63%). As for women who had an abortion, half reported having had it in French Guiana (5/26.31%). Regarding rapid testing for STIs, it was found that a significant portion of women had never had the test (12/63.15%).

As for the professions performed before the migratory process to the mining regions, the most important were paid domestic activity, fisherwoman, janitor, housewife, teacher, farmer, saleswoman, waitress, coal worker and sex worker. The professions performed in the mining environment were cook, *marretei*ra, housewife, housekeeper, freight forwarder (carries the transport of prospectors from Ilha Bela to the mining companies located in French Guiana via the *catraias*), gold prospector, hairdresser. During field observation, it was found that the activities performed by these women in mining can designate tasks that are similar to those carried out in other places, as well as denote other configurations, such as the mining activities themselves. Activities are also flexible between these trades. A cook can also play the role of *marreteira*, or other functions, as long as there is no longer someone responsible for this function in the ravine.

The guiding questions led participants to reflect on the biographical details related to their daily lives and what it is like to be a woman in mining areas from the interaction with peers, which triggered two themes. The first one refers to life before mining, what led them to mining and what they do to obtain resources for self-sustainability in the mining environment, called *Life trajectories: how women deal with in mining*. The second referred to the resources activated by these women in each of the spheres that question each other and their lives as mining workers and mothers, which we categorized as *Woman, mother and prospector: the multiple facets of gender inequality in the Amazonian border*.

### Life trajectories: how women deal with in mining

The women’s search for work in mining was driven by the prospect of changing their lives, motivated mainly by a romantic relationship, both by the invitation of partners who already lived in mining and as an escape from a previous abusive relationship. Other motivators were also mentioned, such as an invitation from friends or family and/or a past marked by domestic violence. After the decision was made to work in mining, women had the option of being recruited through their own travel expenses or through a loan or advance payment, which entails indebtedness prior to mining work.

The life of women in mining promotes intense transformations in their daily lives, considering the rupture with their place of origin and the need to access other internal and external resources to reach the category of mining woman. These women risk going to work in clandestine areas, as they experience a context of extreme poverty. Paradoxically to it, mining is initially presented as an attractive alternative, allowing the hope of sudden enrichment. However, a day in the life in mining gives them another reality, in which it is necessary to make use of other survival strategies. This question contextualizes the precarious social, emotional and structural conditions of women in mining activities, as a scenario of tensions, disputes and power relations.

*I’ve worked in all these places, I’ve been to Tapajós, to Cayenne, to Suriname, to France* [Europe]. *It was a friend of my brother-in-law’s friend who paid for everything* [laughs]. *He has some machines in Suriname. It’s full of money. He buys some cars in Pará to sell there, you know? But I’ll tell you, see? Mon Die, to Suriname, I won’t be back. My ex-husband who paid my bills, if it wasn’t for him, I would still be there today, I had to have sex for money to pay for food, clothes, house! Arriving here, they killed him.* (I3)

Physical and symbolic violence was a transversal point in participants’ lives, especially violence perpetrated by an intimate partnership, either before or after migrating to work in mining. The violence perpetrated by the State, through the condition of clandestinity, was also reported.

*I left Cayenne, then I went to work in the companies that are there. I spent 3 days away from home, when I arrived, they had broken into my house and stolen almost everything. They set it on fire, then I got upset. I have nothing, I have no roof, no floor* [crying]. *I spent 10 years building a house and then he came* [former partner] *and set it on fire.* (I18)

*Violence here is also very complicated, as we are far away, we witness a lot* [...] *Rosa* [fictitious name] *was assaulted by her husband and she told us, she had already been beaten up again. She almost died, then the men here caught him, beat him and kicked him out. He had to go to the hospital. He was very mean to her, said things to let her down.* (I8)

*Those gendarmes* [French police], *if they catch us with gold, they’ll take everything. I mean, they never did anything to me. They already left my husband naked, then they searched everything, then they returned only the clothes and spanked him a lot.* (I6)

In mining, women are challenged to adapt to ensure their survival and security and understand the contractualities established in the place, considering their condition of vulnerability, their relationships with their body, sexuality, as well as the subsistence of their children. In this way, another significant presence of the performances triggered by them was the category that questioned sexuality, work and motherhood.

### Woman, mother and prospector: the multiple facets of gender inequality in the French-Brazilian border

In this sense, in relation to family composition, some women declared not having children. However, when interviewed on later days, they replied that they had had children, but that they lived in other locations. In this way, it is observed that motherhood emerged as a theme of difficult elaboration by women to talk about their children. The experience of mourning the early death of their children in the face of violence that circumscribes mining is referenced in the women’s statements. The conflicts and deaths that occurred in these sites are usually silenced through a kind of coexistence pact.

*They killed in mining, because my son worked with this guy and he said that my son didn’t pay* [the gold], *you understand? He killed him. The brother of the guy who killed him worked with my son. When he called my son to sum up the account* [count the gram of gold], *I know he called my son a thief. He said it was missing 950 grams of gold. They made a trap* [ambush] *for my son. When he went to guard* [the gold], *they called him a thief and killed him. So, that’s why it was like this, because I can’t do anything, right? But they already killed them in another mining there.* (I1)

The report of women who have children revealed that there is a common practice among mothers who experience the routine of working in mining in the region and leaving their children in the urban center. With no family networks close to the municipality, these women entrust the care of their children to third parties, in exchange for grams of gold. Another context observed during the interviews was the concern with child sexual exploitation and the safety of children:

*I don’t bring my daughter here; I leave her there in the city. In Oiapoque, she can study, because these men here are no good, they beat women, they want to steal our things. My girl is 9 years old, you know? There is a lady who takes care of her. I say don’t leave her on the street, because no one trusts these men. Once they get what they want, they don’t want to know anymore, right?* (I17)

*Here, I can get 2 thousand reais and give my children a decent living condition in Oiapoque.* (I6)

The sexual act in exchange for favors, such as a ride to mining, gifts, such as lingerie, food, hygiene products, protection, transport, etc., are considered natural for women. The sexual service, also known as “*ploc*”, was only recognized upon payment in gold or cash in exchange for sex. In the mining areas, they cost an average of 5 grams of gold, which is equivalent to an approximate amount of R$650.00 (about US$130).

Regarding condom use, an improved disposition towards health self-care was observed. However, little negotiating power was also seen with intimate partnership. Despite verbalizing the importance of consistent use of condoms, their use was portrayed in a fortuitous way. The reports intersect the difficulties in negotiating condom use:

*We arrive in the morning so they can take the goods to us* [laughs]. *The men here want everything, because the service is expensive, right?! It costs 5 gram in hand.* (I4)

*Talk to the men here, because they don’t want to use condoms, and I’ve heard that there are about 10 people here with that disease, right?* [whispering AIDS]. (I8)

*My first time, I used a condom, but now I’m together with a person, you know? Then I don’t use it. I know people can’t trust a man. I even like condoms, because you don’t have that stickiness that makes you sick, but we forget, right? When you live together, the men here don’t like to use it either, see?* (I2)

## DISCUSSION

To discuss the results, it was necessary to reflect on the social interactions^(17)^ of women in mining areas. At different moments of interaction, social actors play different roles, given the specific context of mining. In this way, it was sought to draw a sociodemographic profile of women in mining areas and, later, to carry out a problematization of how these women activate mining activities as their main perspective of life.

The predominant profile was that of mature, northern and northeastern, black/brown and functionally illiterate women, similar to another case series about women who earn their living in activities limited to mining^(7)^. The biography of these women intertwines with mining as the only and, sometimes, the last alternative way of life and work they could resort to^(7,24)^, under the motivation of acquiring better living conditions^(3)^. The workforce agency often involves indebtedness prior to the start of work activities, and the costs are paid to their employers later^(2)^.

It is verified that there is transit in different work activities and accumulation of functions and journeys carried out in search of new chances of life, being necessary that they reinvent themselves, playing different roles in each situational context experienced. In mining, as well as in other forms of labor sociability^(25)^, women also receive less than men, even when performing activities of high physical effort^(7,24)^. In this situational context, there is a tension, as the social stigma experienced by women in mining areas attributes them to a condition of subordination, expressed by the difficulty of being fully accepted among other gold workers, due to their condition as women. Thus, there is a tension in the relationships with men who work in mining areas when these women manage to conquer the space to carry out activities understood as eminently masculine, such as the act of looking for and finding gold, considering that this activity is a reason for the recognition of power in the mining society^(7)^. Gradually, these women take over a new role according to the social context experienced: that of a strong, courageous and lucky woman, as she goes beyond the line of “bluffed” men prospectors, thus building a new profile.

As described, in the specific case of mining, it is observed that men are assigned the role of looking for gold ore, while women are assigned activities to maintain the environment organization. In this perspective, the process of stigmatization experienced by these women is present in almost all aspects of mining, as it attributes to women a smaller, pejorative and marginal social place, by giving the functions performed by men in mining a greater social value than the functions performed by women, imposing on them a male hierarchy to the detriment of women^(26)^, which corroborates the perpetuation and naturalization of situations of gender violence.

It is also noteworthy that, for the most part, activities performed by women prior to mining and those performed today converge in jobs that are similar to the concept of sexual division of labor^(25)^. The sexual division of labor is seen in different cultures and societies, which categorize what is work to be done by men and what is work to be done by women, in which, in a materialistic way, the connection of men to the productive sphere, and of women, to the reproductive and care sphere predominates^(25)^.

In the present study, it was observed that intimate partnerships were mentioned as the majority form of recruitment for work. Other studies in mining areas carried out with women corroborate these findings^(7,25)^. Differences in recruitment methods, driven by affective partnerships, demonstrate specificities when it comes to the eminently female context. Geographic isolation and the arduous work routine make it difficult for these women to relate outside the mining environment. Still in this sense, it should be noted that the debts previously acquired by women sometimes assume agreements that involve payment through the sexual act.

Extreme poverty and food insecurity can turn women into forced labor and sexual exploitation in mining. It appears that these women are exposed to the context of extreme poverty and can engage in transactional sex as a source of income and access to basic subsistence goods^(24)^. In this study, women demonstrated not to perceive transactional sex as sexual exploitation. This does not mean to say that they do not reflect on their condition as women in mining, however, the problematization is elaborated from the local sociocultural conceptions or even from the facade they take over as mining women.

But this mining woman can also be a mother, daughter or another category that requires her to have different roles at the time of interaction, based on a certain social context at the time of interaction. This gives rise to the importance of a care plan that considers local cultural values and aspects. This data implies the need for a plan that includes access to information so that these women can understand their rights and the provisions available by the State, given that their profession is a choice and not the only option presented for their livelihood. The choice only exists when there is access to information and assistance from public authorities.

In mining, it is observed the existence of prostitution whose sexual service costs 5 grams of gold, and there are also sexual services to men in exchange for their clandestine assistance, in which women receive groceries for their subsistence and protection. This condition of women was also observed in other informal mining areas in South Africa and other countries in the Amazon region^(7,24)^.

It is known that the practice of unsafe sex, especially in regions characterized by social inequality and reified forms of violence, exposes women with little or no education to STIs. The literature catalogs that, among the predictive factors for STIs among precarious migrants in French Guiana, there are sexual intercourse before age 16, use of alcohol or drugs before sex, and having recently engaged in commercial sex^(27)^.

It is observed that, in these mining, there was a reference to multiple intimate partnerships and between people of different nationalities, suggesting the circulation of STIs between countries. On the Maroni River, a gateway to several clandestine mining sites, located near Ilha Bela, cultural polygyny habits are described in the literature and also correlated in this study^(28)^. Isolation and the consequent decrease in community regulation, poverty and gender-related issues intersect to increase the likelihood that women living in these places will be involved in situations of vulnerability in the sexual and reproductive sphere.

The absence of STI testing presents eminent flaws in women’s sexual and reproductive health care. Such procedures are part of protocols and routines in gynecological, prenatal and childbirth care listed by the Ministry of Health^(29)^. It is noteworthy that the conditions of isolation and reduced access to health services were demonstrated by the expressive number of women without access to rapid testing.

In these spaces, where people live transiting between borders, hidden inside the Amazon Forest, the usual strategies of sexual and reproductive health care are not effective. It is noteworthy that the Brazilian National Policy for Comprehensive Care for Women’s Health (*Política Nacional de Atenção Integral à Saúde da Mulher*)^(29)^ and the Brazilian National Policy on Sexual and Reproductive Rights (*Política Nacional dos Direitos Sexuais e dos Direitos Reprodutivos*)^(30)^ consider the diversity of municipalities and states, which present different levels of development and organization of their local health systems and types of management. Moreover, they encourage joint construction and respect for autonomy, emphasizing the importance of empowering Unified Health System (*Sistema Único de Saúde*) users and their participation in instances of social control.

In this regard, this study sought to give voice to these women, to understand their interaction with the group and with health services, so that, from the gaps explained and observed by them, care can be offered in which they can have really membership and sustained access. The study’s observations have important consequences for communication and prevention strategies and are essential for planning care based on equity.

Health care for women in remote mining areas can be improved through the provision of local or itinerant health services that use rest and support sites, such as Ilha Bela, as a strategy to reach these populations that exercise commuting. Greater efforts of social inclusion and assistance by the Brazilian State must be implemented. There was a lack of information on sexual and reproductive rights. Health education can be a powerful tool for mitigating situations of misinformation and, therefore, greater vulnerability.

### Study limitations

Collection was carried out at the rest station on the Brazilian side of the border, which made it impossible to observe the interaction in the *currutelas* of mining itself. However, despite these limitations regarding security issues due to the participants’ clandestine context, this study allowed to give voice to women who, for a long period, were silenced, on the fringes of public policies of the State.

### Contributions to nursing, health, and public policies

The guarantee of universal and equitable access to health is diametrically related to the reach and use of health services which, in the case of the collaborators, are violated by multiple factors. Improving knowledge about the health of women living in remote areas with high population flows is an important factor for the elaboration of a cross-cultural care plan and the formulation and assessment of social protection policies.

## FINAL CONSIDERATIONS

The daily life of women in the Amazon mining region is characterized by an overlap of vulnerabilities, in which black and brown women with low education promote a rupture with their places of origin, predominantly in the states of the North and Northeast regions, encouraged to work on mining in the border region by intimate partnerships, in the expectation of better living conditions. Mining presents a set of norms and rules of its own, agreed modes of sociability, cultural specificities, being a place of tensions, power struggle and hegemonically masculine, in which women are challenged to perform roles to survive, with a precarious social support network, taking on different facades at the time of interaction.

Violence is expressed, silenced and regulated in everyday life, in which conflicts and murders are perennial, with the experience of mourning, pain, suffering and loneliness of women in the Amazon mining. The sexual and reproductive rights of these women are daily usurped. The female body is a means of subsistence, situations of sexual exploitation, as well as the establishment of multiple intimate partnerships without the consistent use of condoms. Assessing the daily experiences of women in the Amazon mining highlights the need to direct and implement public and social policies and health practices for the comprehensive care of these women’s health.

## References

[1] Sarney J, Costa P. (2004). Amapá: a terra onde o Brasil começa. Senado Federal.

[2] Douine M, Mosnier E, Le Hingrat Q, Charpentier C, Corlin F, Hureau L (2017). Illegal gold miners in French Guiana: a neglected population with poor health. BMC Public Health.

[3] Silva AS, Landim FO. (2017). Conflitos socioambientais entre a comunidade da sede distrital de Vila Brasil, Oiapoque - Amapá e o Parque Nacional Montanhas do Tumucumaque: a fronteira franco brasileira em debate. Rev Eletrôn PRODEMA.

[4] Franco VC, Peiter PC, Carvajal-Cortés JJ, Pereira RS, Gomes MSS, Mutis MCS. (2019). Complex malaria epidemiology in an international border area between Brazil and French Guiana: challenges for elimination. Trop Med Int Health.

[5] Organisation mondiale de Protection de la Nature (2008). Dossier de presse: l’orpaillage illégal en Guyane: fléau majeur pour la forêt, l’eau et la santé humaine [Internet].

[6] Mendes LMC. (2018). Promoção da saúde no contexto fronteiriço.

[7] Freitas LJG. (2016). Mulheres no garimpo: vulnerabilidade do trabalho feminino na Amazônia. Appris;.

[8] Stewart A. G. (2020). Mining is bad for health: a voyage of discovery. Environ Geochem Health.

[9] Cossa H, Scheidegger R, Leuenberger A, Ammann P, Munguambe K, Utzinger J, Macete E, Winkler MS. (2021). Health studies in the context of artisanal and small-scale mining: a scoping review int. J. Environ. Res Public Health.

[10] Mosnier E, Carvalho L, Mahamat A, Chappert J, Ledrans M, Ville M (2015). Épidémies multiples in camps d’orpaillement en forêt amazonienne (Guyane française) en 2013: quelles leçons pour l’accès aux soins et à prévention?. Bol Epidemiol Hebdomadaire [Internet].

[11] Niemetzky F, Mosnier E, Nacher M, Stroot J, Brousse P, Pommier de Santi V (2015). Epidémie de Béri-Béri chez des orpailleurs en Guyane française. Bull Veille Sanit - Cire Antill-Guyane[Internet].

[12] Schwartz FW, Lee S, Darrah TH. A (2021). review of health issues related to child labor and violence within artisanal and small-scale mining. Geohealth.

[13] Wilches-Gutierrez J, Documet P. (2018). What is known about sexual and reproductive health in Latin American and Caribbean mining contexts? a systematic scoping review. Public Health Rev.

[14] Lightfoot E, Maree M, Ananias J. (2009). Exploring the relationship between HIV and alcohol use in a remote Namibian mining community. Afr J AIDS Res.

[15] Luis ST. (2017). Prevalence of HIV at the Kokoyo informal gold mining site: what lies behind the glitter of gold with regard to HIV epidemics in Mali? a community-based approach (the ANRS-12339 Sanu Gundo cross-sectional survey). BMJ Open.

[16] Gonzalez DJX, Arain A, Fernandez LE. (2019). Mercury exposure, risk factors, and perceptions among women of childbearing age in an artisanal gold mining region of the Peruvian. Amazon Environ Res.

[17] Nyanza EC, Joseph M, Premji SS, Thomas DSK, Mannion C. (2014). Geophagy practices and the content of chemical elements in the soil eaten by pregnant women in artisanal and small scale gold mining communities in Tanzania. BMC Pregnancy Childbirth.

[18] Sanna A, Hiwat H, Briolant S, Nacher M, Belleoud D, Le Tourneau FM (2019). Investigation of a possible malaria epidemic in an illegal gold mine in French Guiana: an original approach in the remote Amazonian forest. Malar J[internet].

[19] Hanf M, Bousser V, Parriault MC, Van-Melle A, Nouvellet ML, Adriouch L, Sebillotte CG, Couppie P, Nacher M. (2011). Knowled geoffree voluntary HIV testing centres andwillingnessto do a testamongmigrants in Cayenne, French Guiana. AIDS Care[internet].

[20] Miranda AE, Merçon-de-Vargas PR, Corbett CEP, Corbett JF, Dietze R. (2009). Perspectives on sexual and reproductive health among women in an ancient mining area in Brazil. Rev Panam Salud Publica. [Internet].

[21] Goffman E. (1992). A representação do eu na vida cotidiana.

[22] Vinuto J. (2014). A amostragem em bola de neve na pesquisa qualitativa: um debate em aberto. Temáticas [Internet].

[23] Minayo MCS. (2014). O desafio do conhecimento: pesquisa qualitativa em saúde.

[24] Jenkins K. (2014). Women, mining and development: an emerging research agenda. Extr Ind Soc.

[25] Melcher EJG, Oliveira MHB, Carvalho RME. (2017). Violência doméstica e trabalho: percepções de mulheres assistidas em um Centro de Atendimento à Mulher. Saúde Debate.

[26] Sharma S, Suran R. (2007). Consideration of the determinants of women’s mental health in remote Australian mining towns. Austral J Rural Health.

[27] Eubanks A, Parriault MC, Van Melle A, Basurko C, Adriouch L, Cropet C (2018). Factors associated with sexual risk taking behavior by precarious urban migrants in French Guiana. BMC Int Health Hum Rights.

[28] Cobat A1, Halfen S, Grémy I. (2008). Determinants of condom use and heterosexual multiple sexual partnership in French Antilles and French Guiana. Rev Epidemiol Sante Publique.

[29] Ministério da Saúde (BR) (2013). Saúde sexual e saúde reprodutiva.

[30] Ministério da Saúde (BR) (2005). Direitos Sexuais e Direitos Reprodutivos: uma prioridade do governo.

